# Dr. Alexander Hugh Ferguson

**Published:** 1911-12

**Authors:** 


Dr. Alexander Hugh Ferguson of Chicago, whose partnership with Dr. Monahan, we noted last month, died of diabetes October 20, 1911, aged 58. He was a Canadian, an honor graduate of Trinity, 1881.

